# Validating the Malaysian modified checklist for autism in toddlers, revised with follow-up (M-CHAT-R/F): a cross-cultural adaptation

**DOI:** 10.3389/frcha.2023.1221933

**Published:** 2023-08-02

**Authors:** Yung Lin Han, Wan Shahrazad Wan Sulaiman, Abdul Rahman Ahmad Badayai, Hilwa Abdullah @ Mohd. Nor

**Affiliations:** Faculty of Social Sciences and Humanities, National University of Malaysia, Bangi, Malaysia

**Keywords:** autism spectrum disorder, Malaysia, screening tool, M-CHAT-R/F, validation

## Abstract

**Introduction:**

The Modified Checklist for Autism in Toddlers, Revised with Follow-Up (M-CHAT-R/F) is a two-stage parent-reported tool for screening autism spectrum disorder (ASD). Early detection of ASD is highly associated with improved social communication and reduced restricted and repetitive behaviors associated with ASD. However, there is limited availability of ASD screening tools in Malaysia and there are no relevant validation studies published. The process of modifying a screening instrument to align with the cultural and linguistic characteristics of the target population is a crucial component in establishing the instrument's validity.

**Methods:**

Therefore, this study translates and culturally adapts the M-CHAT-R/F into Malay and verifies its psychometric properties among the Malaysian population. 500 Malaysian toddlers aged between 18 and 48 months were recruited from different settings. The parents of the toddlers were asked to complete the Malaysian M-CHAT-R/F. The reliability of the screening tool was verified using Cronbach's alpha.

**Results:**

By comparing the screening outcomes of the Malaysian M-CHAT-R/F and clinical evaluation results, the prevalence of ASD was determined as 6.6% in the sample. High values of sensitivity (96.6%) and specificity (93.2%) and a satisfactory positive predictive value (47.5%) supported the validity of the Malaysian M-CHAT-R/F. Furthermore, the receiver operating characteristic analysis yielded three as the optimal cut-off score of the Malaysian M-CHAT-R/F.

**Discussion:**

These results suggest that the Malaysian M-CHAT-R/F is an effective screening tool reliable for use in clinical practice. Further investigation using a representative sample of the whole country is recommended given the high prevalence rate obtained in the current sample.

## Introduction

Autism Spectrum Disorder (ASD) is one of the most common neurodevelopmental disorders. It is characterized by impaired social interactions and communication, and repetitive and restrictive behaviors (1, 2). The global prevalence of ASD has dramatically increased over the years (3–8). According to the Autism and Developmental Disabilities Monitoring Network, the prevalence of ASD is reported at 1.85%, where one in 54 children in the United States is diagnosed with ASD (7). Since the epidemiology of ASD remains unknown, its screening and diagnosis heavily rely on subjective qualitative criteria (9), such as questionnaires and behavioral assessments. The American Academy of Pediatrics has recommended using broadband screening in toddlers' healthcare visits at 18 and 24 months to detect those with high chance of ASD (10). However, a 2009 survey shows that only 28% of pediatricians perform the recommended routine screening, specifying that the most common barriers are lack of time and lack of familiarity with screening tools (11). Typically, ASD screening tools are parent-reported questionnaires that can be completed either online or in primary healthcare settings in less than 10 min (12).

Among the available screening tools, Robins et al.'s (13) Modified Checklist for Autism in Toddlers, revised with follow-up (M-CHAT-R/F) is often used to detect autism-related symptoms in toddlers. It is identified as a level one screening tool as it can be administered by non-professionals in general settings (14) to provide an initial identification of subsequent needs for diagnosis and intervention. In fact, this revised version was developed to improve the utility of its predecessor, M-CHAT (15). The clinical utility and validity of a screening tool is often evaluated by reporting its sensitivity and specificity values. Sensitivity value refers to the test's ability to accurately identify samples with a condition, while the specificity value refers to the ability to identify samples without the said condition (16). Sometimes, the Positive Predictive Value (PPV) which refers to the probability that a received positive test result would mean actual diagnoses (17), were also reported to provide additional validation measures. Using a low-risk population as sample, the M-CHAT-R/F reported moderate sensitivity (66.7%) and high specificity (99.5%). In addition, the M-CHAT-R/F was claimed to have better utility as the authors reported higher PPV (51%) as compared to the previous. A higher PPV is often favored as it could reduce unnecessary anxiety and further reduce the needs for additional tests.

However, language and cultural differences hinder the maximum utilization of parent-reported screening tools (18), particularly in non-Western cultures where English is not the primary language. Although studies have shown a high degree of universality in the early symptom presentation of ASD across populations (19–21), the screening accuracy results often differed significantly. For example, the sensitivity and specificity of the M-CHAT-R/F were reported to be different across translation and validation studies that have been conducted on different populations (22–25). Moreover, the positive predictive value (PPV) reported were also differed significantly, ranging from 8.6% to 100% (26, 27). Although the differences in the PPV can be explained by its high dependence on the prevalence rate of the disorder, the differences in sensitivity and specificity warrant careful consideration if the adapted versions are equally effective when administered in other populations (28).

Notably, most studies that examine the validation and cross-cultural adaptation of the M-CHAT-R/F have not reported the details of their translation process (26, 29, 30). This casts further doubt on the reliability and validity of such screening tools, as previous research has shown that extensive translation and adaptation efforts often result in the instrument containing more modified or culturally appropriate phrases (31). In fact, only a few studies examining cross-cultural adaptation and validity of the M-CHAT-R/F have had little modifications in wording or examples (24, 25, 32). Another inconsistency observed, is the age range of the participants (26, 30, 33, 34). Despite the M-CHAT-R/F being suggested to screen toddlers from 16 to 30 months, some studies have reported to include subjects up to the age of 48 months. Although studies with such an extended age range have reported high sensitivity and specificity (20), it is difficult to reach a conclusion if the screening tool is as effective when used among older toddlers with limited statistical data. In addition, it is suggested that cross-cultural differences might lead to under- or over-recognition in reporting somatization of symptoms (35)*.* This further highlights the importance of cultural adaptation in the application of translated instruments.

In Malaysia, approximately 50% of ASD cases are identified after the age of five (36), due to low public awareness about ASD and the lack of professional workforce in clinical settings (37, 38). Currently, Malaysia still uses the previous version, M-CHAT (39), to identify young toddlers with high likelihood of ASD. The M-CHAT was translated into Malay and incorporated into routine heath care visits for all children at 18 and 36 months respectively (40). However, there were no validation studies on the translated M-CHAT (36), nor its clinical application in Malaysia. In fact, the translated Malay version of M-CHAT in the Children Health Record book was initially published by Lau et al. (36) to study the efficacy of M-CHAT in differentiating between ASD with other developmental disorders instead of being a cross-cultural validation study of M-CHAT. Moreover, no study has examined the prevalence of ASD among Malaysians, nor have there been any efforts to validate a broadband screening tool.

Hence, this study translates, culturally adapts, and validates the Malaysian M-CHAT-R/F and examines its sensitivity and specificity. Although the present study was not aimed to be an epidemiology study, however, as the participants were recruited from different community settings and the sample size recruited is large, the results of this study may provide a preliminary representation of the general population. Therefore, the results of this study also present a projection of prevalence rate of children with ASD in Malaysia; apart from the PPV and optimal cut-off score of the Malaysian M-CHAT-R/F. In addition, cross-cultural adaptation of a screening instrument for an intended population is also an integral part of establishing validity. As a result, the research's findings would further contribute to the validity assessment of M-CHAT-R/F. Considering that the target population's culture and language play a crucial role in establishing an instrument's cross-cultural validity, this study refers to Tsang et al.'s (41) translation and adaptation processes. In addition, several similar adaptation studies of M-CHAT-R and M-CHAT-R/F (20, 25, 32) for other populations were also referred for methodological approach.

## Methods

### Study design and settings

This current research adopts a prospective approach, and the data collection procedures were integrated into the existing healthcare services. Participants were recruited from different settings, specifically four community clinics, two hospitals, four pre-schools, and three therapy centers, in Selangor. Taking into considerations that most community clinics in Malaysia has long waiting list with limited professional workforce, some parents might opt to have their child's developmental screening done in private centers with relevant medical specialist. The three therapy centers recruited are private medical therapy centers which offer community developmental screenings for general populations around the area. Hence, all participants are recruited from the general population. According to the official portal of the Ministry of Economy of Malaysia (42), Selangor was reported to have the highest number of households (*N* = 1,623.1). It is placed as the third highest in monthly household gross income (RM 10,827) and monthly household consumption (RM 4,709), as well as third lowest in absolute poverty (1.2%) among the 14 states of Malaysia. In addition, it is also reported that a mean monthly household gross income for the top 20% of Malaysian is equivalent to RM 18, 506, middle 40% is RM 7,348 and the bottom 40% is RM 3,152. In terms of population by ethnic group, majority of Malaysian are Malay (69.9%), followed by Chinese (22.8%), Indian (0.06%) and others (<0.01%).

Therefore, though detailed demographic data could not be obtained due to ethical reasons as compliance to the approval given by the Ministry of Health Malaysia, through review of economic statistical published for Selangor, the subjects recruited in this research are projected to be from the middle-upper class in the urban area.

### Participants

Based on the calculation using Krejcie and Morgan's (43) formula, the required sample size for a target population of 100,000 and above is 384. With a 20% dropout rate (44) for most research studies, the required sample size for the first phase of this study was determined at a minimum of 461 subjects. Only toddlers aged between 18 and 48 months with an identified caregiver who spoke or read Malay, and provided written informed consent at the time of screening were included. In addition, as this study aims to validate the M-CHAT-R/F through analysis of sensitivity and specificity values as well as PPV; only children who has not been through developmental screenings are included to closely resembles the practicality of M-CHAT-R/F being a broadband screening tool. Any toddlers who do not fit the age range, or those who had been clinically diagnosed with other medical conditions or developmental disorders were excluded as the aim of this study is not to evaluate the efficacy of M-CHAT-R/F in differentiating children with various disorders or syndromes compared to ASD. Children with previously diagnosed ASD were also excluded as this study took on a prospective approach rather than retrospective.

**Figure 1:**
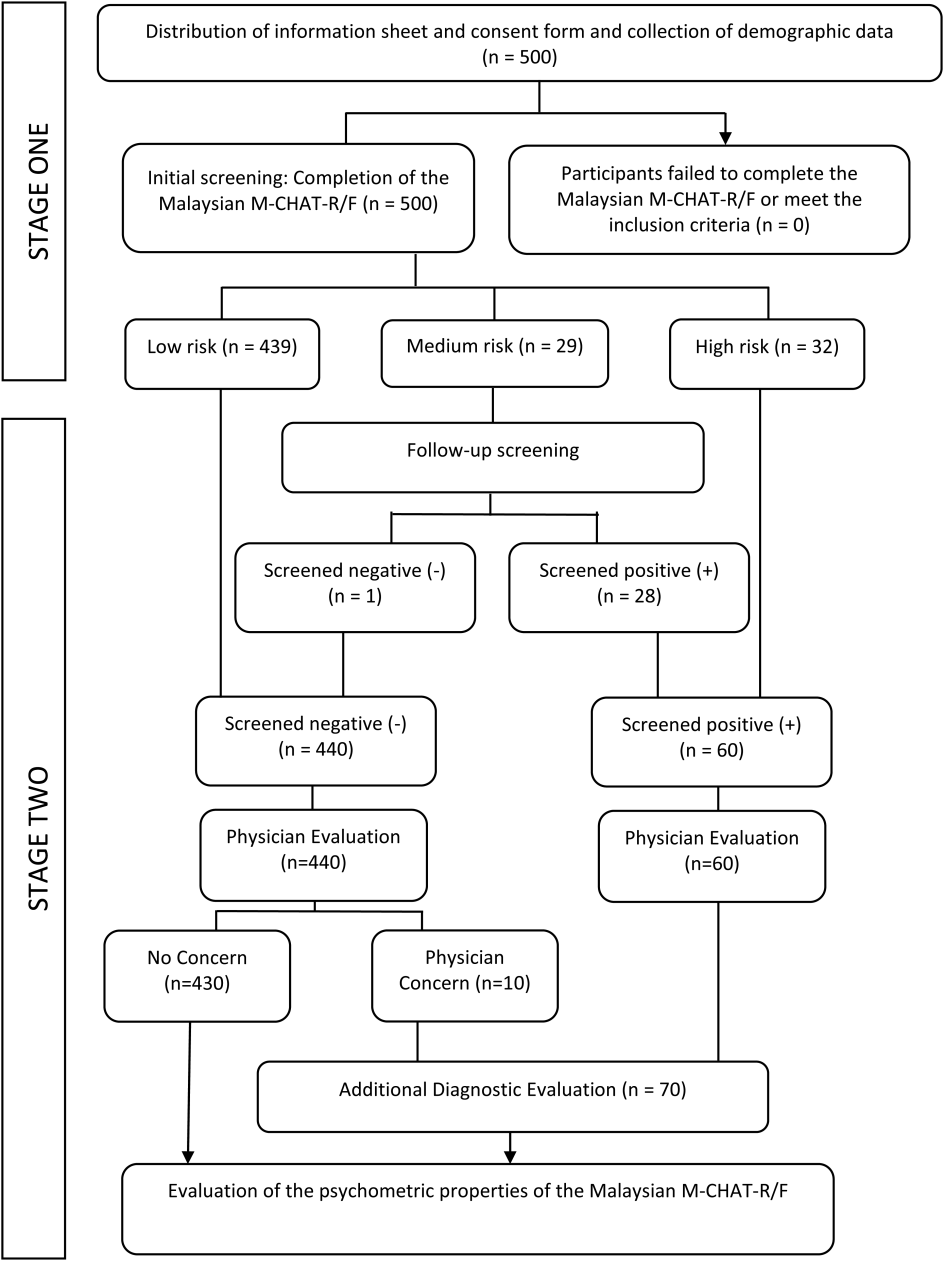
Initial and follow-up screening procedures to validate the Malaysian M-CHAT-R/F.

The final sample comprised of 500 toddlers, with a mean age of 27.92 months (SD = 8.20). Most toddlers were males (53.6%) and aged between 18 and 30 months (79.4%). Data of most toddlers were collected at community clinics (78.0%), followed by schools (10.4%), therapy centers (8.4%), hospitals (2.6%), and other platforms (0.6%). The other platforms consisted of social media platforms, such as parenting and early childhood education groups on Facebook, and orphanages. Table 1 presents the descriptive statistics of participants' demographics and Figure 1 illustrates the flow of screening procedures.

**Table 1:** Participants’ demographics (*N* = 500).

Characteristic	
Age in months at the screening, mean (SD)	27.92 (8.2)
Age group, *n* (%)	
18–30 months	397 (79.4)
31–48 months	103 (20.6)
Sex, *n* (%)	
Male	268 (53.6)
Female	232 (46.4)
Race, *n* (%)	
Malay	280 (56.0)
Chinese	137 (27.4)
Indian	81 (16.2)
Others	2 (0.4)
Location of data collection, *n* (%)	
Community Clinic	390 (78.0)
Hospital	13 (2.6)
School	52 (10.4)
Therapy Center	42 (8.4)
Others	3 (0.6)

### Measures

#### The Malaysian M-CHAT-R/F

**Figure 2:**
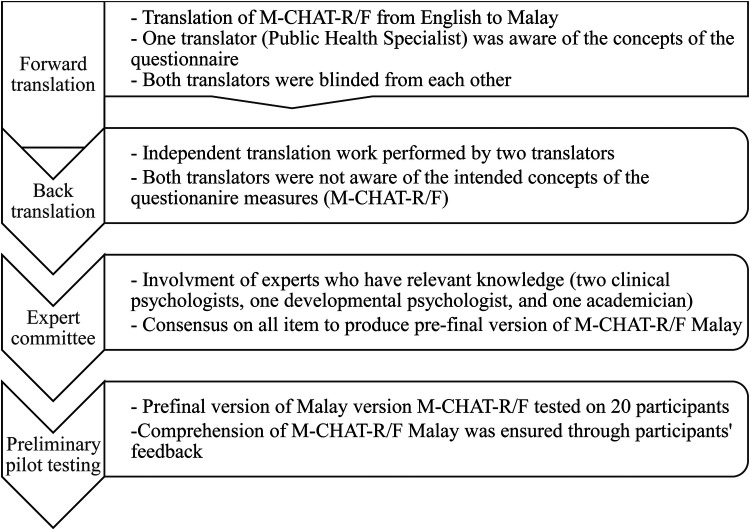
Translation procedure of M-CHAT-R/F adapted from Tsang et al. (41).

Permission to translate the M-CHAT-R/F into Malay was obtained from the original authors. According to Tsang et al.'s (41) guidelines, the four steps in the translation and adaptation process are forward translation, back translation, establishment of an expert committee, and preliminary pilot testing, as shown in Figure 2. The English M-CHAT-R/F was forward-translated into Malay by personnel from the Ministry of Health who also translated the previous version (M-CHAT Malay) and a pediatrician. An expert committee comprising two clinical psychologists, one developmental psychologist, and one academician compared the English and translated versions to resolve any discrepancies. Discrepancies include the use of relevant words and examples. For example, the phrase “*bermain berlakon atau olok-olok*” was used to represent play pretend or make-believe, which is not a direct translation of the English version but a Malay phrase which carries the same meaning and widely use among the community. Other examples are usage of “*respons*” and “*snek*” which are borrowed words from the English instead of using the actual Malay translation as these borrowed words were more widely used among the community in daily lives. Upon agreeing to the forward translation, the Malay M-CHAT-R/F was back-translated into English by a linguistic expert and a bilingual academician who were blinded to the purpose and concepts of the screening tool. Any discrepancies were resolved by the expert committee. A preliminary pilot test of the Malaysian M-CHAT-R/F was conducted with a small sample of 20 bilingual Malaysian parents. No incongruities or ambiguities were observed. In fact, nurses and parents who participated in the pilot test commented that the examples included in most of the items were very helpful as compared to the previous M-CHAT that does not provide any examples. The final Malaysian M-CHAT-R/F comprised 20 yes–no questions with three reverse-scoring items.

#### Clinical assessments

Clinical assessments include preliminary evaluation conducted by pediatrician or family medicine specialist during initial healthcare visits, and diagnostic evaluation conducted by licensed clinical psychologists who had at least five years of experience. Preliminary evaluation was based on semi-structured parent interviews, Ages and Stages Questionnaires—Third Edition (45), Schedule of Growing Skills-II (46), or the Bayley Scales of Infant and Toddler Development—Third Edition (47), as well as the criteria listed in Diagnostic and Statistical Manual-5 (1). Depending on the child's presentation and the physician's recommendations during initial health care visits, additional assessments, such as the Autism Diagnostic Observation Schedule—Second Edition (48) and the Wechsler Preschool and Primary Scale of Intelligence—Third Edition (49), were also performed by the clinical psychologists as second level diagnostic evaluation. Children who fulfilled the criterion of ASD throughout both evaluations would receive a diagnosis of ASD and grouped under “ASD”, while children were screened negative and did not exhibit specific autistic characteristics during physician evaluations would be grouped as “non-ASD” in this research.

### Procedures

The Institutional Review Board of the National University of Malaysia provided oversight and approval for this study (JEP-2021-871). Additionally, external ethical approval (NMRR ID-22-00626-8LB) was obtained from the Ministry of Health Malaysia, namely the Medical Review & Ethics Committee and the National Medical Research Register, before recruiting participants from community clinics and hospitals. Registered preschools and therapy centers were approached individually for recruiting participants. The researchers provided an informed consent form, information sheet, and the Malaysian M-CHAT-R/F questionnaire to the medical staff and teachers working at the recruitment sites. The medical staff and teachers were instructed about inclusion and exclusion criteria so that they could screen children aged between 18 and 48 months for this study. As such, parents were invited by these professionals and briefed about the nature of this study by presenting the information sheet. Parental consents were obtained as they signed on the informed consent form. Then, the Malaysian M-CHAT-R/F were given to the parents and subsequently checked by these professionals or researchers for any missed items. Follow-up screenings were done through phone calls or on-the-spot interviews.

The Malaysian M-CHAT-R/F is a two-stage ASD screening tool for toddlers aged between 18 and 48 months. The first stage consists of a checklist containing 20 yes–no questions, while the second involves a structured follow-up interview. Between April 2022 and December 2022, the Malaysian M-CHAT-R/F was used to screen 500 Malaysian toddlers aged between 18 and 48 months. In the first stage, parents or caregivers responded to 20 questions related to specific autistic traits based on their child's developmental performance. The scores were summed, but Items 2, 5, and 12 were reverse scored. A total score between zero and two, three and seven, and eight and twenty denoted low, medium, and high risk of ASD, respectively. Children who scored two or less were considered to have screened negative, while those who scored more than two were considered to have screened positive in the first stage. The children who had screened negative (those having a low likelihood of ASD) were not subjected to a follow-up interview. On the other hand, since children with medium risk had scored more than two in the first stage, their parents or caregivers were invited for a follow-up telephonic or on-the-spot interview and were asked about failed items. If the child continued to screen positive after the follow-up interview (that is, the child failed two or more items), the child was referred for an additional clinical diagnostic evaluation.

As the screening occurred and was integrated into healthcare visits, physicians were asked to determine whether ASD features were present in all children despite the M-CHAT-R/F scores acquired. As practicing physicians in current study were equipped with knowledge and practical experience to diagnose ASD based on its criterion and performed preliminary evaluation of ASD as part of their healthcare services, children who did not exhibit any related features were regarded as non-ASD at this stage. Meanwhile, ten children who were initially screened negative were flagged by these physicians for presence of ASD features and the parents were invited for subsequent clinical diagnostic evaluation. Children with high risk were directly considered to have screened positive in the second stage (without a follow-up interview) and were referred for additional clinical diagnostic evaluation. The final diagnosis of ASD was concluded based on all gathered information inclusive of assessment results and clinical judgment according to the DSM-5.

There were no incomplete responses, hence all responses are included for statistical analyses. Descriptive statistics were used to determine participants' demographics. The reliability of the Malaysian M-CHAT-R/F was assessed using Cronbach's alpha, where a value greater than 0.70 was considered adequate (50). The screening results were compared with evaluation results (ASD vs. non-ASD), and the prevalence of ASD was calculated. The validity of the Malaysian M-CHAT-R/F was determined based on its sensitivity, specificity, and PPV in accordance with the full sample of 500 participants. Its clinical validity was determined for the target age group (toddlers aged between 18 and 48 months) as well as segregated age groups (toddlers aged between 18 and 30 months and 31 and 48 months). We investigated its validity for segregated age groups, especially the 31–48 age group, to gather evidence of its efficacy in detecting ASD in older toddlers. Finally, the optimal cut-off score of the Malaysian M-CHAT-R/F was determined in relation to the Malaysian population using the receiver operating characteristic (ROC) analysis. All statistical analyses were performed using SPSS version 25 at a significance level of *α* = 0.05.

### Statistical analysis

Several statistical procedures were performed using the SPSS v27 to speculate the psychometric performances of the Malaysian M-CHAT-R/F. Descriptive analysis for each item, inclusive of comparison between children who were diagnosed with ASD after screening and those without is presented. Normality test was not performed as the nature of the screening scores would be positively skewed as majority of the subjects recruited were from the general populations with presumably low M-CHAT-R scores, representing the majority portion of neurotypical children. Hence, reliability of the Malaysian M-CHAT-R/F was assessed based on its' internal consistency of the test items using Cronbach's Alpha as all the 20 items were measuring the same construct. Then, the validity of the Malaysian M-CHAT-R/F was evaluated based on its' sensitivity, specificity and PPV by reviewing and comparing the scores and assessments results of the 500 samples recruited. In addition, regression analysis was administered to demonstrate items predictive power on the actual diagnosis. The optimal cut-off score was also calculated using the Receiver Operating Characteristic (ROC) curve.

### Community involvement statement

The family members of children with autism were involved in reviewing the translated screening tool and provided feedback for the expert committee, especially during the pre-test of the translated M-CHAT-R/F. Parents mentioned that the examples given were helpful and regarded them as an added advantage compared to the previous version. Doctors, nurses, and parents of children from the autistic community were also involved in sharing their subjective experiences and thoughts while filling the self-administered Malaysian M-CHAT-R/F. Comments mostly includes ease of administration and accuracy of examples depicting the daily lives and Malaysian cultures.

## Results

### Descriptive statistic

Descriptive analysis for each item of the Malaysian M-CHAT-R/F was computed. Table 2 showed the frequencies and percentage of children in each group who scored at risk on each item. Overall, the highest scored item is item no. 5 with *N* = 66. The highest scored item (*N* = 45) among non-ASD participants is item no. 5 while the highest score items (*N* = 38) among ASD participants are item no. 7, 18 and 16.

**Table 2:** Frequencies and percentage of children in each group who scored at risk on each item*.*

Frequencies and percentage of scoring 1 (at risk)	Total (*n* = 500)	Non-ASD (*n* = 440)	ASD (*n* = 60)
	n (%)	n (%)	n (%)
1. If you point at something across the room, does your child look at it?	23 (4.6)	0 (0)	23 (38.33)
2. Have you ever wondered if your child might be deaf?	25 (5)	13 (2.9)	12 (20)
3. Does your child play pretend or make-believe?	32 (6.4)	5 (1.13)	27 (45)
4. Does your child like climbing on things?	24 (4.8)	10 (2.27)	14 (23.33)
5. Does your child make unusual finger movements near his or her eyes?	66 (13.2)	45 (10.23)	21 (35)
6. Does your child point with one finger to ask for something or to get help?	38 (7.6)	4 (0.9)	34 (56.67)
7. Does your child point with one finger to show you something interesting?	42 (8.4)	4 (0.9)	38 (63.33)
8. Is your child interested in other children?	33 (6.6)	1 (0.23)	32 (53.33)
9. Does your child show you things by bringing them to you or holding them up for you to see—not to get help, but just to share?	29 (5.8)	1 (0.23)	28 (46.67)
10. Does your child respond when you call his or her name?	24 (4.8)	2 (0.45)	22 (36.67)
11. When you smile at your child, does he or she smile back at you?	25 (5)	2 (0.45)	23 (38.33)
12. Does your child get upset by everyday noises?	57 (11.4)	41 (9.32)	16 (26.67)
13. Does your child walk?	8 (1.6)	4 (0.9)	4 (6.67)
14. Does your child look you in the eye when you are talking to him or her, playing with him or her, or dressing him or her?	31 (6.2)	3 (0.68)	28 (46.67)
15. Does your child try to copy what you do?	26 (5.2)	3 (0.68)	23 (38.33)
16. If you turn your head to look at something, does your child look around to see what you are looking at?	49 (9.8)	11 (2.5)	38 (63.33)
17. Does your child try to get you to watch him or her?	42 (8.4)	5 (1.14)	37 (61.67)
18. Does your child understand when you tell him or her to do something?	39 (7.8)	1 (0.23)	38 (63.33)
19. If something new happens, does your child look at your face to see how you feel about it?	40 (8)	4 (0.9)	36 (60)
20. Does your child like movement activities?	14 (2.8)	2 (0.45)	12 (20)

### Reliability

The Cronbach's *α* was 0.91, showing a high level of internal consistency across all 20 items of the Malaysian M-CHAT-R/F. According to George and Mallery (51), this value denotes excellent internal consistency. The inter-item correlation for all 20 items was within the ideal range, with a mean value of 0.34 (52). Table 3 showed the changes in Cronbach's alpha if the item is deleted. Since there was no significant increment in reliability after an item was deleted, we retained all 20 items.

**Table 3:** Item-Total statistics.

Item	Cronbach's Alpha if Item Deleted
1	.906
2	.918
3	.908
4	.915
5	.922
6	.907
7	.906
8	.905
9	.906
10	.908
11	.908
12	.921
13	.917
14	.906
15	.908
16	.906
17	.906
18	.903
19	.905
20	.913

### Screening outcomes

After the completion of the Malaysian M-CHAT-R/F, most participants (88.0%) screened negative. In the first stage, most participants (87.8%) had a low risk of ASD, followed by those who had a high risk (6.4%) and those who had a medium risk (5.8%). In the second stage, medium-risk participants who had failed two or more items and those with high risk were considered to have screened positive. As shown in Table 4, 440 participants screened negative, while 60 screened positive after the second stage. Participants who were identified to fulfil the criterion of ASD based on clinical assessments are classified as “ASD” while participants who do not fulfill the criterion throughout the screening procedures are classified as “non-ASD”.

**Table 4:** Screening outcomes after the Use of the Malaysian M-CHAT-R/F.

Risk of ASD	*n* (%)
Low	439 (87.8)
Medium	29 (5.8)
High	32 (6.4)
Screened Negative of ASD	440 (88.0)
Screened Positive of ASD	60 (12.0)
ASD	33 (7.5)
Non-ASD	407 (92.5)

### Prevalence of ASD

Although determining the prevalence of ASD among Malaysian children was not the main intention of this study, it could be calculated by comparing the screening and diagnostic outcomes. Since the participants were recruited from different settings and none were lost during the follow-up interview and healthcare screenings by physicians, the prevalence calculated in this study may serve as a preliminary findings and projection on the prevalence among the Malaysian population. According to Figure 1, 70 children were referred for additional clinical diagnostic evaluation. 32 out of 60 children who screened positive received a diagnosis of ASD, while only 1 out of 10 children who screened negative but had a physician concern received a diagnosis of ASD after subsequent clinical diagnostic evaluation. Thus, 33 out of 500 recruited toddlers received a diagnosis of ASD, suggesting the prevalence rate of ASD amounted to 6.6%.

### Validity of the Malaysian M-CHAT-R/F

Table 5 shows that the Malaysian M-CHAT-R/F demonstrates decent sensitivity and specificity at 97.0% and 94.0%, respectively, and a satisfactory PPV of 53.3% among Malaysian toddlers aged between 18 and 48 months. It also presents the results for younger and older age groups. The sensitivity and specificity values were nearly the same for both age groups. The screening accuracy for the younger age group (18–30 months) was slightly more precise (sensitivity = 100.0%, specificity = 94.2%, and PPV = 42.1%) than that for the older age group (31–48 months) (sensitivity = 94.1%, specificity = 93.0%, and PPV = 72.7%). However, the PPV for the older age group was 30.6% more than the PPV for the younger age group. In addition, Table 6 shows the predictive power for each item on the actual diagnosis. The table shows that the Malaysian M-CHAT-R/F is statistically significant in predicting the actual diagnosis, *F*(20, 249) = 114.51, *p *< 0.001. Multiple regression showed that 11 out of 20 items in the Malaysian M-CHAT-R/F instrument are significant predictors of actual ASD diagnosis, *p *< 0.05.

**Table 5:** Sensitivity, specificity, and positive predictive value of the Malaysian M-CHAT-R/F.

	Sensitivity (95% CI)	Specificity (95% CI)	PPV	NPV	FP	FN
M-CHAT-R/F^a^	97.0%	94.0%	53.3%	99.8%	6.0%	3.0%
M-CHAT-R/F^b^	100.0%	94.2%	42.1%	100.0%	5.8%	0
M-CHAT-R/F^c^	94.1%	93.0%	72.7%	98.8%	7.0%	5.9%

CI: confidence interval; PPV: positive predictive value; NPV: negative predictive value; FP: false-positive; FN: false-negative; M-CHAT-R/F: Modified Checklist for Autism in Toddlers, Revised with Follow-up; M-CHAT-R/F^a^: toddlers aged between 18 and 48 months; M-CHAT-R/F^b^: toddlers aged between 18 and 30 months; M-CHAT-R/F^c^: toddlers aged between 31 and 48 months.

**Table 6:** Predictive Power for Each Item on the Actual Diagnosis

Item	β	SE	95% CI		*p*
			Lower Bound	Upper Bound	
If you point at something across the room, does your child look at it?	.01	.04	−.06	.10	.678
Have you ever wondered if your child might be deaf?	−.04	.02	−.09	.00	.054
Does your child play pretend or make-believe?	−.01	.03	−.06	.04	.658
Does your child like climbing on things?	.03	.03	−.02	.09	.190
Does your child make unusual finger movements near his or her eyes?	.01	.02	−.02	.04	.616
Does your child point with one finger to ask for something or to get help?	.16	.03	.09	.20	<.001
Does your child point with one finger to show you something interesting?	.12	.03	.05	.17	<.001
Is your child interested in other children?	.45	.04	.38	.52	<.001
Does your child show you things by bringing them to you or holding them up for you to see—not to get help, but just to share?	−.14	.03	−.21	−.08	<.001
Does your child respond when you call his or her name?	−.02	.03	−.08	.05	.569
When you smile at your child, does he or she smile back at you?	.24	.04	.19	.34	<.001
Does your child get upset by everyday noises?	−.02	.02	−.05	.01	.235
Does your child walk?	−.01	.04	−.09	.06	.690
Does your child look you in the eye when you are talking to him or her, playing with him or her, or dressing him or her?	−.05	.04	−.12	.02	.182
Does your child try to copy what you do?	.06	.03	.00	.13	.043
If you turn your head to look at something, does your child look around to see what you are looking at?	−.07	.03	−.11	−.01	.019
Does your child try to get you to watch him or her?	.18	.03	.10	.22	<.001
Does your child understand when you tell him or her to do something?	.06	.04	−.02	.13	.120
If something new happens, does your child look at your face to see how you feel about it?	.10	.03	.04	.15	.002
Does your child like movement activities?	−.10	.03	−.22	−.09	<.001

### Optimal cut-off score of the Malaysian M-CHAT-R/F

**Figure 3:**
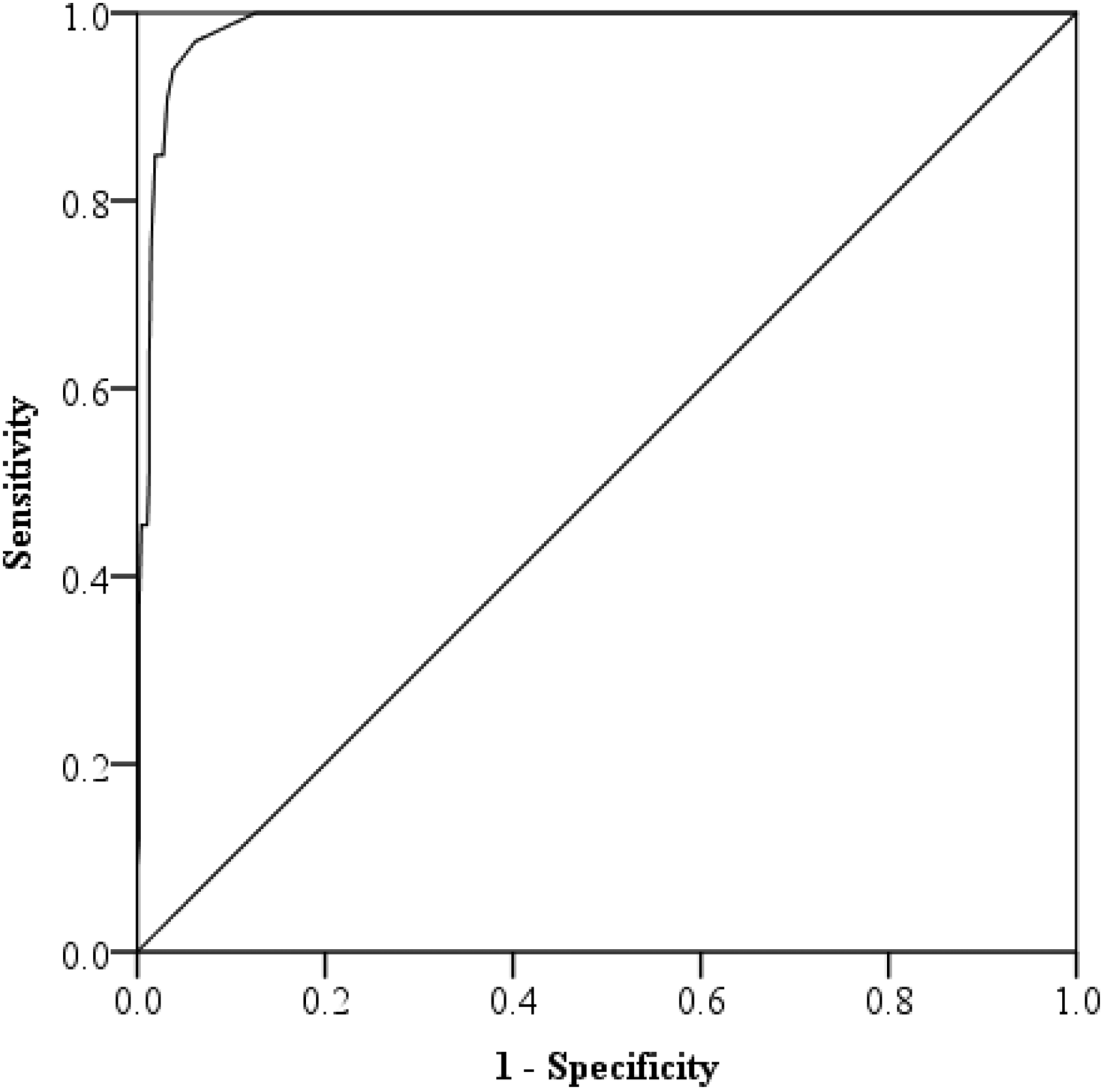
Receiver operating characteristic (ROC) curve of the Malaysian M-CHAT-R/F.

We used the ROC curve to determine the optimal cut-off score of the Malaysian M-CHAT-R/F. According to Swets (53), when the Area Under the Curve (AUC) is closer to one, it indicates that the test has better discriminability. Figure 3 shows that the AUC was 0.986, indicating that the Malaysian M-CHAT-R/F has relatively good discriminability in accurately distinguishing ASD. Furthermore, after maximizing sensitivity without compromising specificity, where the predictive values revealed a sensitivity of 0.97 and a specificity of 0.94, we found that the optimal cut-off score was three. This cut-off score corresponded to the cut-off score of the original M-CHAT-R/F, as shown by Robins et al. (13).

## Discussion

We analyzed the psychometric properties of the Malaysian M-CHAT-R/F using extensive translation and adaptation processes and administering it to a sample of 500 Malaysian children aged between 18 and 48 months. The results showed that the tool has excellent reliability, with its Cronbach's *α* (0.91) being higher than that of the original version of the M-CHAT-R/F (0.79) (13). In addition, the high predictive values of sensitivity (0.97) and specificity (0.94) and the cut-off scores of the Malaysian M-CHAT-R/F were consistent with those found in the initial validation study with American children (13) and other adaptation studies with Indonesian (30), Chinese (24), and Icelandic children (54). The presence of high sensitivity and specificity values indicates that the Malaysian M-CHAT-R/F is highly effective in detecting ASD characteristics and features among children, and differentiating them from those who are neurotypical, providing evidence of its efficacy as a screening tool. In fact, more than 90% of the toddlers who screened positive in the first stage of the Malaysian M-CHAT-R/F continued to screen positive in the second stage. This finding further showed that the Malaysian M-CHAT-R/F is a promising screening tool that parents can self-administer without having to attend a follow-up interview with nurses or other healthcare professionals. This would undoubtedly help reduce the time spent in healthcare consultations and address the issue of the lack of professional workforce (37, 38). On a side note, parents and nurses commented the examples for each item of the Malaysian M-CHAT-R/F gave a clearer idea and made it easier to give a definite response. It is interesting that no study, inclusive of the initial revised study by Robins et al. (13) commented on the advantages of adding examples to the items questioned.

To the best of our knowledge, this is the first study to segregate the target age group into younger and older age groups, while determining the sensitivity, specificity, and PPV of the M-CHAT-R/F, as well as examine its validity. According to the above-presented results, there were no significant differences between the results obtained for the younger (18–30 months) age group and older (31–48 months) age group. Our results also showed that the Malaysian M-CHAT-R/F is effective for screening older toddlers in Malaysia, similar to how the M-CHAT-R/F was effective in screening older toddlers in Indonesia (30) and Singapore (20). This is also the first study in Malaysia to investigate the efficacy of a screening tool for older toddlers. It is anticipated that having a screening tool with a larger age range will reduce the strain of physicians and practitioners and enhance the healthcare system, particularly in Malaysia's rural areas where healthcare services may not be easily accessible.

Given that the prevalence of ASD differs across regions and most validation studies have not reported the prevalence rate, the PPV is often regarded as a validation measure. In comparison to the PPV (50.9%) found in the initial validation trial (13), this study showed a PPV of 53.3% in the current sample. However, different M-CHAT-R/F validation studies have presented different PPVs, with Sangare et al. (27) reporting the highest PPV (100%) in Mali's sample and Oner and Munir (26) reporting the lowest PPV (8.6%) in Turkey's sample. Since the PPV heavily depends on the prevalence rate (55), recruiting samples from healthcare settings where there may be more toddlers with a higher likelihood of ASD than the general population, may have resulted in bias and contributed to the unstable data across studies (56). For example, when the sample includes a higher proportion of children with high likelihood of ASD, the PPV is higher. Hence, studies that have recruited a large number of participants from the general population have generally reported a lower PPV than those that have recruited participants solely from healthcare settings (26, 33). Since we recruited samples from different settings and used a population-based sample, the lower PPV in this study (53.3%) would seem to be a realistic representation of the occurrence of ASD in the Malaysian population. We also suggest that the PPV should be used as an indicator of an instrument's validity only when the recruited samples closely resemble the general population of the target region.

Interestingly, despite having a lower PPV and recruiting samples from the general population, out of the 500 toddlers we recruited, 33 were diagnosed with ASD in clinical diagnostic evaluations. The prevalence of ASD of the Malaysian sample recruited in this study was then found to be 6.6%, which is much higher than the prevalence in the United States (1.85%) (7) and the global prevalence (approximately 0.62%) (8). However, it is noteworthy that epidemiological studies on the prevalence of ASD in the United States were conducted on children aged eight years and not on young toddlers. Moreover, given that this study was not an epidemiological study, the prevalence rate it presents mainly serves as a guideline. Nevertheless, as this study recruited participants from different settings in Malaysia, the high prevalence among young toddlers is concerning. In fact, we found that there are long waiting lists (a minimum of 12 months) for a consultation slot for a child's developmental diagnosis at government hospitals. This phenomenon may explain Lau et al.'s (36) finding that approximately 50% of ASD cases in Malaysia are diagnosed after the age of five. Therefore, future research to investigate the prevalence of ASD using a representative sample of the whole country is highly recommended.

This study holds great significance as it details how the Malaysian M-CHAT-R/F was translated and culturally adapted based on Tsang et al.'s (41) guidelines. Although the website of the M-CHAT-R/F's author has more than 40 translations (https://mchatscreen.com/mchat-rf/translations; Retrieved 3rd October 2022), many of the translation and adaptation studies have either not been published or most do not explain the translation and adaptation processes. According to a recent systematic review (18), translated screening instruments may not be psychometrically equivalent to the original instrument due to the language and cultural differences. It has also been found that performing more quality assurance measures during translation and adaptation processes generally results in higher-quality instruments (57). Hence, one of the most prominent strengths of this study is that it highlights the importance of such steps and offers a higher possibility of replication. Based on Tsang et al.'s (41) guidelines, we invited professionals from relevant fields as translators, formed an expert committee to resolve discrepancies, and performed a preliminary pilot test before conducting the main study. Although it is not fully data driven, this study serves as a valuable guideline for future translation and adaptation studies.

Furthermore, a notable aspect of this study is its effective incorporation into community healthcare practices during the data collection phase. The preliminary evaluation to identify autism-related characteristics was made possible through the participation of the physicians with relevant expertise from the Ministry of Health Malaysia. The utilization of multiple developmental screenings by these physicians during routine healthcare visits enhanced the accuracy of both screening and diagnosis results. In consequence, this approach facilitated the detection of false negatives without the need for comprehensive diagnostic evaluations, which would be impractical given the limited availability of personnel and assessment resources. On the other hand, with the additional step of administering clinical diagnostic evaluations, the true positive rate obtained is presumed to be more reliable. As large-scale low-risk studies often have limitations in assessing screen negative cases given the constraints of conducting numerous evaluations within a limited timeframe (24), the procedures implemented in this study can be considered as an additional illustration to address this issue.

Despite its utility, this study has some limitations. First, not all recruited samples underwent full clinical diagnostic evaluations where relevant ASD assessments were administered. Although physicians were involved to participate and administered preliminary assessment during the routine health checks, true sensitivity and specificity cannot be determined until all samples undergo the full clinical diagnostic evaluations. Moreover, according to previous studies (58, 59), high-functioning children with milder developmental delays may be missed during screening but later diagnosed with ASD. Hence, results from the present findings should be interpreted with caution. Second, this study focused only on the psychometric properties and predictive value of the Malaysian M-CHAT-R/F in screening toddlers for ASD, and not for any other developmental disorders or delays. A handful of toddlers who had screened negative were subsequently diagnosed with speech delay, but this data was not reported or analyzed in this study. Furthermore, due to practical limitations such as lack of professional workforce, limited assessment tools and restrictions in information obtained as part of Malaysia's national ethical practice, in-depth analysis could not be performed. Hence, future research could investigate how the Malaysian M-CHAT-R-/F scores would perform in relation to other variables, such as parent's social-economic statues, IQ scores or other assessment test results. In addition, by referring to Lau et al. (36), future studies may also evaluate how the Malaysian M-CHAT-R/F would perform in differentiating ASD from other developmental disorders. Finally, given that Malaysia has several states and this study only recruited samples from a single state, Selangor, the results presented in this study might not be an accurate representation of the whole population.

## Conclusion

This study shows that the Malaysian M-CHAT-R/F has relatively high sensitivity, specificity, and PPV in screening Malaysian toddlers aged between 18 and 48 months for ASD. Its results provide evidence of the M-CHAT-R/F as a promising population-based screening tool. Moreover, the translation and adaptation processes performed in this study can serve as a guideline for similar future studies. The efficacy of the Malaysian M-CHAT-R/F in screening a larger age range of toddlers for ASD can ease toddlers’ transition from screening to diagnosis, and subsequently enrolment in early interventions which would bring significant positive outcomes.

## Data Availability

The raw data supporting the conclusions of this article will be made available by the authors, without undue reservation.
